# Disease and management beliefs of elderly patients with rheumatoid arthritis and comorbidity: a qualitative study

**DOI:** 10.1007/s10067-018-4167-2

**Published:** 2018-06-09

**Authors:** Marloes van Onna, Betül Öztürk, Mirian Starmans, Ralph Peeters, Annelies Boonen

**Affiliations:** 1 Department of Medicine, division of Rheumatology, Maastricht University Medical Center, PO Box: 5800, 6202 AZ Maastricht, The Netherlands; 2 Department of Rheumatology, Zuyderland Hospital, Henri Dunantstraat 5, 6419 PC Heerlen, The Netherlands

**Keywords:** Comorbidity, Elderly patients, Qualitative research, Rheumatoid arthritis

## Abstract

To explore in elderly patients with rheumatoid arthritis (RA) and comorbidity (1) in which order and why patients prioritize their morbidities with regard to functioning and health, (2) their beliefs about common (age-related) musculoskeletal complaints, and (3) experiences about the influence of comorbidity on medication treatment of RA. Patients between 50 and 85 years with RA and ≥ 1 comorbidity or lifestyle risk factor were invited for a semi-structured interview. Two readers coded the transcripts of the interviews, by using NVivo11 software. Fifteen patients (14 women; mean age 67 years (range 51–83 years); mean disease duration 14 years (range 1–39 years)) were interviewed. Only 3 (20%) out of 15 patients prioritized RA over their comorbidity; these patients often experienced severe functional limitations. The level of current or (perceived) future disability, risk of dependency, and the perceived lethality of a condition were considered by participants when prioritizing morbidities. Most participants had misconceptions about common age-related musculoskeletal complaints. Consequently, these participants attributed all joint complaints or even all physical complaints to RA, disregarding degenerative joint disease and physiological aging as alternative diagnoses. Half of the participants ever had to change RA medication because of comorbidity. Most of these patients had prioritized the comorbidity, sometimes even over treatment of RA disease activity. Most elderly RA patients with comorbidity prioritize the importance and treatment of comorbidity over RA. Better understanding of patients’ beliefs on RA and comorbidity is essential when managing chronic conditions in elderly patients.

**Electronic supplementary material:**

The online version of this article (10.1007/s10067-018-4167-2) contains supplementary material, which is available to authorized users.

## Introduction

The incidence and prevalence of rheumatoid arthritis (RA), a chronic inflammatory autoimmune disease that primarily affects the joints, increases due to population aging and improved survival of patients [1]. Patients with RA are also, due to the chronic inflammation associated with RA, at increased risk of (accelerated) development of comorbidity including cardiovascular disease, malignancies, and osteoporosis. Currently, the average patient with RA has two or more comorbid conditions [2]. Moreover, aging by itself also predisposes to the development of comorbidity.

The management of RA in elderly patients is challenging, since the benefit of treatment should always be evaluated against the comorbid burden and the risk of doing harm due to medication side effects. Several studies described the phenomenon of age bias in the treatment of elderly RA patients. Age bias refers to the observation that elderly patients receive less intensive treatment compared to younger patients [3]. In a study by Kremers et al., disease modifying anti-rheumatic drugs (DMARDs) were initiated in younger RA patients at a significantly earlier time point (hazard ratio (HR) per 10-year decrease in age 1.4; 95%-confidence interval 1.3–1.5) when compared to elderly patients [3]. Even after controlling for the level of disease activity and number of comorbidities, rheumatologists still preferred the less intensive treatment option in elderly patients. Yet, it is not clear which factors contribute to age bias. For instance, rheumatologists might “adjust” for degenerative joint disease, such as osteoarthritis (OA). On the other hand, RA patients with comorbidity and polypharmacy are likely to have their own beliefs and convictions about the different diseases and medications they have to deal with [4]. At this moment, there is limited knowledge about the disease and management beliefs of elderly RA patients. The objectives of this present qualitative study were (1) in which order and why elderly patients prioritize their morbidities with regard to functioning and health, (2) to explore beliefs about common (age-related) musculoskeletal complaints, and (3) explore the experiences about the influence of comorbidity on medication treatment of RA.

## Methods

### Study design and participants

For this qualitative study, elderly patients with RA were invited for one semi-structured interview with a duration of approximately one to one-and-a-half hour. Rheumatologists from one academic and one large non-academic clinic in the south of The Netherlands were asked to recruit eligible patients. RA patients aged between 50 and 85 years who have ≥ 1 comorbidity and/or ≥ 1 lifestyle risk factor were invited to participate. Patients with severe cognitive impairment were excluded. Recruitment stopped after enrollment of a group of patients that reflected the complete age range and until theoretical saturation was reached (i.e., the framework could not be further extended with new information). The interviews were conducted by one interviewer (BO, 6th year female medical student) in March and April 2017. Prior to the study commencement, there was no relationship established between the interviewer and interviewee. The information obtained during the interview was not communicated to the treating rheumatologist. The study was approved by the institutional review board from the Maastricht University Medical Center. All participants provided written informed consent.

### Data collection

To secure uniform data quality and comparability, an interview guide was developed that included both open-ended and closed questions. A pilot interview was conducted to train the interviewer and to ensure that all questions were clear and addressed all important topics. All interviews were audio recorded and afterwards fully transcribed.

After recording age, disease duration, and general medical history, the interviewer addressed the following topics with the patient (more detailed information: Table 1):

- Beliefs about common (age-related) musculoskeletal complaints, such as OA.
- In which order and how patients prioritized their morbidities with regard to functioning and health.
- Whether RA medication ever had to be adjusted because of another condition; if yes, did the patient prioritize treatment of the RA or the comorbidity and why?

**Table 1:** Most important questions derived from the interview guide to explore patients’ beliefs regarding aging and development and prioritization of comorbidity

Questions about RA and aging in general	
What do you think about aging in general? What makes it difficult? Are there also advantages? Are there joint complaints that you consider to be age-related? Are you able to differentiate these complaints from RA-related complaints? If so, how do you do that?	
Questions about RA and comorbidity	
Can you tell me about the other medical conditions you have, apart from RA? How many doctors do you visit, apart from your GP? Which medical condition takes most of your time? Why? About which medical condition do you worry the most / the least? Why? Is it possible for you to prioritize your medical conditions? Which condition is the most ‘important’ one, when you consider the impact on your health and daily functioning? Why? Which condition is the least ‘important’ one? Why? Does comorbidity influence the medical treatment of your RA? If yes, can you give an example?	
Questions about medication treatment for RA	
Does it ever happen to you that you receive conflicting advice from different medical specialists? Can you give an example? When related to medication for a specific medical condition, which advice do you follow? Do you then prioritize the medication for RA or the comorbidity?	

Questions are translated from Dutch

*RA* rheumatoid arthritis, *GP* general practitioner

As the interviewer was allowed to ask supplementary questions to further explore the views of the participants, the interview guide was not exhaustive.

Comorbidities were collected during the interview and confirmed by chart review. From these data, the Rheumatic Disease Comorbidity Index (RDCI) was computed [5]. Functional and disability status was calculated using the Health Assessment Questionnaire Disability Index (HAQ-DI) that was filled out by patients after the interview (duration 20 min) [6].

### Data analysis

A grounded theory-influenced approach was used to ensure a systematic process was followed in developing knowledge and theory [7]. Once an interview was completed, the audio recordings were transcribed and transferred to a qualitative data analysis software package (NVivo 11). Data were analyzed anonymously. Initial results guided subsequent data collection. Themes (i.e., recurrent unifying concepts or statements) and subthemes were identified from the study data and illustrative quotes made by patients were collected. Data were constantly compared and passages were re-read until no new (sub)themes were uncovered. Next to coding, text passages were also commented in order the guide the process of textual interpretation. All transcripts were read, annotated, and analyzed by the first reader (BO). The second reader (MO) also read the interviews and checked whether all constructs and statements had been identified and linked to themes and subthemes. The readers regularly met to discuss interpretation of the data. In case of disagreement, consensus was reached between the two readers after re-reading the specific passage of the transcript.

Results of the RDCI and HAQ-DI were computed by using descriptive statistics.

## Results

### Participant characteristics

Fifteen patients with ≥ 1 comorbidity and/or ≥ 1 lifestyle risk factor agreed to participate and were interviewed. A short description of the included patients can be found in Supplementary Table 1. The most frequent comorbidities were osteoporosis (six patients), type 2 diabetes mellitus (five patients), OA (four patients), and cardiovascular disease (three patients). The three topics formulated beforehand (Table 1) were further explored during the interview. A new theme that emerged during the interviews was etiology of RA and/or comorbidity. Each theme could be underpinned by several subthemes, mostly influential factors, such as polypharmacy. The main themes are discussed below, including supporting quotes.

With regard to aging in general, most patients did not specifically worry about their RA. Instead, cognitive decline and social dependency were often mentioned as a worrying factor (quote 1). However, most patients also appreciated the fact that they have more leisure time to spend with family and friends and experience less work-related stress.

Quote 1: “I hope that I will get old, provided the fact that I am still the same person. No dementia or things like that, in that case I don’t want to get older.” *Patient 5*, *64 years old.*

### Prioritization and accommodating chronic conditions

Of the 15 patients, only three patients prioritized RA over their comorbidity. The level of current and/or (perceived) future disability attributed to a chronic condition, the perceived risk of dependency, and the perceived lethality of a condition were important factors when prioritizing. “Asymptomatic” risk factors such as hypertension and hypercholesterolemia were less often a health concern. Comorbidity most often prioritized over RA included cardiovascular disease, ophthalmologic conditions, malignancies, osteoporosis, diabetes mellitus, and depression. As an example, two out of six patients with osteoporosis prioritized osteoporosis as most important. These two patients had a fragility fracture in their medical history and expressed worries about disability in case of new fracture. Patients with an ophthalmologic condition often prioritized this as the most important, because they were anxious about dependency due to visual loss (quote 2). Several patients expressed RA is “less important” because RA is not a lethal condition as opposed to cardiovascular disease or a malignancy (quote 2–4).

Quote 2: “You can adapt to RA. If you have a problem with your eyes, you can’t adapt. (...) My eyes are priority no. 1.” *Patient 11*, *51 years old.*

Quote 3: “I think that I worry the most about my breast cancer. This is actually life threatening if not treated properly. Yes, because I feel, if it is not treated well, then the survival chances are small of course. With RA, at least as far as I know, you will not die from it. RA is a very annoying, but you are not dying from it.” *Patient 12*, *52 years old.*

Quote 4: “Well, my heart consumes most of my time, from a psychological viewpoint. When your heart does not work well, it can kill you. RA does not kill you.“ *Patient 2*, *60 years old.*

For most patients, RA was the first chronic disease they had to cope with. Many patients expressed that they adapted to RA. New comorbidity then became more of a worry, also because this comorbidity (for instance type 2 diabetes mellitus) was a risk factor for developing additional comorbidity, such as cardiovascular disease (quote 5).

The three patients who prioritized RA over their other chronic diseases highlighted the fact they experienced many restrictions during daily life activities due to RA and that RA management consumed a lot of their time (quote 6). Two out of these three patients also had a higher than average HAQ score as compared to the patients who did not prioritize RA as most important.

Quote 5: “Yes, the RA is important, I have it already for many years. And now I get diabetes as a second disease. And, then the GP tells me: “you have RA, you have diabetes, now you are a so-called risk patient.” (...) “For heart attacks, all kinds of diseases”. *Patient 15*, *69 years old.*

Quote 6: “Yes, my whole life is about RA. RA, you will never get rid of it. RA is part of my life for the last 40 years.” *Patient 5*, *64 years old.*

### Beliefs about common age-related musculoskeletal complaints

Thirteen (87%) patients had misconceptions about common age-related musculoskeletal complaints, such as OA (quote 7). Even patients, who have had joint replacement surgery due to OA, were not aware of a difference between OA and RA-related complaints (quote 8). Twelve (75%) patients related all their musculoskeletal complaints to RA, and some patients related even all their physical complaints, including fatigue and general weakness to RA (quote 9 and 10), disregarding alternative diagnoses such as OA or aging. When exploring why patients related most complaints to RA, participants responded that they experienced many unassignable complaints, which makes it difficult to differentiate (quote 9). Since RA is the disease they are most familiar with, the complaints are assigned to RA or medication prescribed for RA (quote 10). Also, patients responded that they do not “know better because they have had RA for years, what else could it be?” (quote 11). Two patients were aware of the difference between OA and RA. One patient indicated she had asked her rheumatologist about the cause of her joint complaints and had been explained the difference in symptoms related to OA compared to RA. The other patient could make a differentiation based on her personal experience: “RA is associated with swollen, warmer and stiffer joints”. Remarkably, almost none of the patients spontaneously related musculoskeletal complaints to aging.

Quote 7: “The doctor calls it osteoarthritis. But look at these joint nodules.”

Interviewer: “And what do you think?”

“For me is everything RA. Yes, I do not think that getting older is associated with the appearance of joint nodules. When you have RA for 14 years, then you are focused on RA.” *Patient 9*, *76 years old.*

Quote 8: “Can osteoarthritis become worse after a diagnosis of RA? I do not know. Osteoarthritis, I do not have it anymore. Now, that all belongs to RA.” *Patient 8*, *73 years old.*

Quote 9: “I always link that to RA. I shrunk 7 centimetres, look how I sit. I link everything to RA.” *Patient 13*, *73 years old.*

Quote 10: “Yes, if I had no RA, I would be able to differentiate (*i.e. between physical complaints*). RA itself, when you read the medication brochures, it is all about being tired, blurred vision, all those complaints...” *Patient 13*, *73 years old*.

Quote 11: “I link all my joint complaints to my RA.”

Interviewer: “Why?”

“Yes, why? Well, a few years ago I did not have any of these complaints. I have no idea what else can cause these complaints, no idea.” *Patient 6*, *83 years old*.

### Experiences about influence of comorbidity on medication treatment of RA

Seven patients worried about polypharmacy. These seven patients had on average three comorbidities, and they expressed that they wanted to use as few medications as possible. Patients mainly worried about medication interactions, since interactions may cause new side effects, such as nausea or hypertension. One patient admitted that she refused to take prednisone, even at the cost of higher RA disease activity (quote 12).

About half of the participants ever had to adjust their RA medication because of comorbidity. The majority of these patients prioritized treatment of the comorbidity over the treatment of RA (quote 13). Despite the fact that patients often prioritized types of medication, they expressed to be equally adherent for the medication prescribed for those chronic conditions that they perceive as less important.

The majority of patients shared beliefs about the etiology of their RA and/or comorbidity. These patients attributed their day-to-day physical complaints or comorbidity to RA or to the medication prescribed for RA. These assumptions were sometimes correct (e.g., steroid-induced diabetes mellitus or hypertension due to leflunomide), but in some cases, the link between the complaint/comorbidity and RA was less clear (e.g., blurry vision due to methotrexate). Prednisone often appeared to be “the malefactor” (quote 12). Participants expressed that side effects did not affect their medication adherence.

Quote 12: “I still think that prednisone is the malefactor. I just do not want it. Yes prednisone, you get a moonface. You feel very unhappy.” *Patient 1*, *67 years old.*

Quote 13: “First I used naproxen with methotrexate. At the pharmacy, they told me that naproxen is not good for my heart. I told this to my rheumatologist and I had to make a choice. And I honestly chose for my heart*.” Patient 2*, *60 years old.*

## Discussion

To the best of our knowledge, this is the first qualitative study that focuses on the disease and management beliefs of elderly patients with RA and comorbidity. Most participants in our study prioritized other chronic conditions over RA. Almost all patients in our study had misconceptions about common (age-related) musculoskeletal complaints, such as OA. As a result, almost all joint complaints were attributed to RA. Of the patients who ever had to change their anti-rheumatic medication due to comorbidity, the majority preferred the treatment of the comorbidity over the treatment of RA.

With regard to aging in general, patients in our study often worried about dependency. This finding is in line with a study of Buitinga et al., in which five future worst-care scenarios were presented to 74 RA patients [8]. The scenario “being dependent on others” was chosen by 35% of RA patients as the worst to experience. However, our study suggests that patients do not necessarily worry about dependency because of RA but are also concerned about dependency due to for instance cognitive decline.

Previous studies also found that patients with several chronic conditions prioritize their conditions [9, 10]. RA is generally seen as a leading cause of disability. However, even when reflecting about disability as a health priority, the vast majority of participants did not prioritize RA, but other comorbidities, as a major concern. As several participants had RA for years, it is conceivable that these patients adjusted to RA and perceive emerging comorbidity as more important. Moreover, patients might feel confident that RA can be treated adequately and RA became less of a worry. Also, RA was not considered a lethal disease by participants. Inadequate management of RA due to other priorities can however lead to (accelerated) development of comorbidity such as cardiovascular disease and consequently premature death. Improving knowledge and addressing common misperceptions about the consequences of RA might change the beliefs of patients about RA and the consequent development of several comorbidities.

The majority of patients had misconceptions about age-related musculoskeletal complaints, such as OA. These misconceptions may in turn lead to poor differentiation between complaints. Several studies found that both aging and comorbidity may independently alter commonly used RA-specific outcome measures, including joint scores, remission, and response criteria and functional disability assessments [11, 12]. For instance, Sokka et al. concluded that only 15% of the general population > 50 years old meet all four ACR remission criteria [11]. Also, in a study by Radner et al. in 380 patients with established RA, it was found that activities of daily living represented by HAQ are equally affected by comorbidities [13]. These findings suggest that defining remission and functioning is more difficult in the elderly patient population, due to other interfering factors such as OA. Hypothetically, rheumatologists might “downgrade” the result of the outcome measure (i.e., lower DAS28 score than actually measured), resulting in correct or incorrect adjustment of anti-rheumatic therapy. In addition, several patients in our present study linked all their physical complaints, including fatigue and general weakness, to RA. These physical complaints are however not only common in patients with RA but can also be viewed as age- or comorbidity-related. Calibrating measures for disease activity and HAQ against the effect of aging as seen in population subjects might be a solution to differentiate between age- and disease-related complaints as a rheumatologist.

Patients in our study prioritized their conditions but expressed to be equally adherent for the medication prescribed for chronic conditions that they perceive as less important. This finding is in contrast to the findings of Rifkin et al., where participants tended to prioritize their medication based on their perceived importance of the condition [14]. Only when RA medication had to be changed due to another condition, the majority of the participants in our study preferred the treatment of the comorbidity. Furthermore, about half of the patients explicitly expressed that it is important to take as few medications as possible, sometimes even at the cost of higher disease activity. Similar perspectives were found in a qualitative study by Van Tuyl et al., which discussed the concept “remission” with 47 RA patients [15]. Patients not only defined remission as absence of symptoms but merely as a decreased impact of their condition on daily life and the feeling of a return to normality. As patients in the study of van Tuyl were younger (mean age 56 years, range 29–76), it is possible that our elderly patients with comorbidity value “acceptable functioning” and “normality” as more important than “absence of symptoms.” Our findings also suggest that the concept of age bias seems not only to arise from the rheumatologists’ point of view; elderly patients can have their own treatment beliefs that lead to age bias.

Several limitations of this study need to be addressed. The study sample was not fully balanced as there was a disproportionate inclusion of women. Generalizability is therefore limited to women under the care of a rheumatologist. However, theoretical saturation was reached (i.e., the data framework could not be further extended). In addition, the scores of for the RDCI and HAQ-DI were low, which indicates that patients had a relatively favorable functional status. Future research therefore needs to focus on elderly patients with higher levels of comorbidity and disability, as these management beliefs might differ from the beliefs of the patients included in our study.

In conclusion, elderly RA patients with comorbidity have their own disease and management beliefs that must be considered when delivering care and developing management goals. This study underlines the need for more effective patient education about aging, RA, and comorbidity. Understanding patients’ beliefs on RA and comorbidity and a shared view about treatment goals should be key priorities when managing RA.

## Electronic supplementary material

Supplementary Table 1 (DOCX 14 kb)
