# Supplementary material for: Disease and management beliefs of elderly patients with rheumatoid arthritis and comorbidity: a qualitative study
Source: Clin Rheumatol. 2018 Jun 9;37(9):2367–72. doi: 10.1007/s10067-018-4167-2 (PMC6097103; doi:10.1007/s10067-018-4167-2)
Supplement: Supplementary file 1 — (DOCX 14 kb) [file 10067_2018_4167_MOESM1_ESM.docx]

Supplementary table 1. Short description of included patients.

| **Patient** | **Description** |
| --- | --- |
| 1 | Female, 67 years old, low level of education, married, RA for 37 years. Anti-rheumatic treatment: hydroxychloroquine, biological. Medical history and comorbidity: osteoporosis, hip fracture. Lifestyle risk factors: hypertension, hypercholesterolemia. HAQ-DI 0.8, RDCI 2. |
| 2 | Female, 60 years old, medium level of education, married, RA for 14 years. Anti-rheumatic treatment: methotrexate. Medical history and comorbidity: myocardial infarction, depression, knee replacement, osteoporosis. Lifestyle risk factors: hypertension, hypercholesterolemia. HAQ 0.3, RDCI 4. |
| 3 | Male, 81 years old, low level of education, married, RA for 13 years. Anti-rheumatic treatment: leflunomide. Medical history and comorbidity: coronary artery bypass graft, type 2 diabetes mellitus. Lifestyle risk factor: hypertension. HAQ-DI 0.3, RDCI 4. |
| 4 | Female, 79 years old, low level of education, married, RA for 13 years. Anti-rheumatic treatment: biological. Medical history and comorbidity: osteoporosis, multiple fragility fractures, malignancy gastro-intestinal tract. Lifestyle risk factor: none. HAQ 0.3, RDCI 2. |
| 5 | Female, 64 years old, medium level of education, married, RA for 39 years. Anti-rheumatic treatment: NSAID, prednisone, sulfasalazine. Medical history and comorbidity: osteoporosis, fragility fracture, pulmonary complaints. Lifestyle risk factor: hypertension and hypercholesterolemia. HAQ-DI 1.9, RDCI 3. |
| 6 | Female, 83 years old, medium level of education, married, RA for 7 years. Anti-rheumatic treatment: prednisone, methotrexate. Medical history and comorbidity: otherwise unremarkable. Lifestyle risk factor: hypertension. HAQ-DI 0.6, RDCI 1. |
| 7 | Female, 63 years old, medium level of education, single, RA for 5 years. Anti-rheumatic treatment: leflunomide, biological. Medical history and comorbidity: type 2 diabetes mellitus, depression. Lifestyle risk factors: none. HAQ-DI 1.4, RDCI 2. |
| 8 | Female, 73 years old, low level of education, married, RA for 1 year. Anti-rheumatic treatment: prednisone, methotrexate. Medical history and comorbidity: severe OA. Life style risk factors: none. HAQ-DI 0.9, RDCI 0. |
| 9 | Female, 76 years old, low level of education, married, RA for 14 years. Anti-rheumatic treatment: methotrexate. Medical history and comorbidity: myocardial infarction, atrial fibrillation. Lifestyle risk factors: hypertension. HAQ-DI 0.4, RDCI 3. |
| 10 | Female, 57 years old, low level of education, married, RA for 1 year. Anti-rheumatic treatment: methotrexate. Medical history and comorbidity: knee replacement, fractures, depression, peptic ulcer, migraine. Lifestyle risk factor: hypertension. HAQ-DI 1.8, RDCI 4. |
| 11 | Female, 51 years old, low level of education, married, RA for 22 years. Anti-rheumatic treatment: NSAID, methotrexate, hydroxychloroquine, biological. Medical history and comorbidity: glaucoma, cataract, type 2 diabetes mellitus, tuberculosis, osteoporosis, depression. Lifestyle risk factor: hypertension, hypercholesterolemia. HAQ-DI 0.3, RDCI 4. |
| 12 | Female, 52 years old, medium level of education, married, RA for 1 year. Anti-rheumatic treatment sulfasalazine. Medical history and comorbidity: breast cancer, OA. Lifestyle risk factor: none. HAQ-DI 0.9, RDCI 1. |
| 13 | Female, 73 years old, medium level of education, married, RA for 16 years. Anti-rheumatic treatment: methotrexate, hydroxychloroquine. Medical history and comorbidity: OA, cataract, hypothyroidism, hyperparathyroidism. Lifestyle risk factors: hypertension. HAQ-DI 1.3, RDCI 2. |
| 14 | Female, 61 years old, medium level of education, married, RA for 4 years. Anti-rheumatic treatment: methotrexate. Medical history and comorbidity: knee replacement, OA, type 2 diabetes mellitus. Lifestyle risk factors: hypercholesterolemia. HAQ-DI 1.4, RDCI 1. |
| 15 | Female, 69 years old, low level of education, widow, RA for 17 years. Anti-rheumatic treatment: gold salts. Medical history and comorbidity: type 2 diabetes mellitus, peripheral arterial disease, osteoporosis, peptic ulcer. Lifestyle risk factors: hypertension, hypercholesterolemia. HAQ-DI 0.4, RDCI 4. |

Abbrevation: RA, rheumatoid arthritis; NSAID, non-steroidal anti-inflammatory drug; OA, osteoarthritis; HAQ-DI (range 0-3), Health Assessment Questionnaire Disability Index; RDCI (range 0-9), Rheumatic Disease Comorbidity Index.
